# Vaginitis in pregnancy is related to adverse perinatal outcome

**DOI:** 10.12669/pjms.313.6752

**Published:** 2015

**Authors:** Fengqiu Xu, Xiaodong Du, Lili Xie

**Affiliations:** 1 Fengqiu Xu, Department of Obstetrics, Lishui Maternity and Children Hospital, Lishui, 323000, P. R. China; 2 Xiaodong Du, Department of Obstetrics, Lishui Maternity and Children Hospital, Lishui, 323000, P. R. China; 3 Lili Xie, Department of Obstetrics, Lishui Maternity and Children Hospital, Lishui, 323000, P. R. China

**Keywords:** Infection, Reproductive tract, Adverse perinatal outcome, Vaginitis, Perinatal mortality

## Abstract

**Objective:**

To determine whether education level and occupation are risk factors of vaginitis in pregnant women and to investigate relationship between vaginitis occurrence during pregnancy and perinatal mortality rates.

**Methods:**

A total of 319 women of early pregnancy or mid-pregnancy were enrolled. Six specimens were collected from posterior fornix of each pregnant woman and then cultured for identification of *Neisseria gonorrhoeae*, intestinal bacteria, general bacteria, fungi, mycoplasma, and chlamydia, respectively.

**Results:**

The pregnant women in the “elementary school or below” group and the “middle school” group had significantly higher incidences of vaginitis compared with the pregnant women in the groups of “high school”, “skill education”, and “college or above”. The pregnant women in the groups of “Worker”, “Government employee”, “Company employee”, and “Professionals” had significantly lower vaginitis incidences. The women with infections of *Neisseria gonorrhoeae*, intestinal bacteria, and general bacteria had higher perinatal mortalities (0.063 ± 0.011, 0.052 ± 0.012, and 0.017 ± 0.008, respectively) than women with infections of fungi, mycoplasma, and Chlamydia (0.002 ± 0.007, 0.003 ± 0.004, and 0.001 ± 0.001, respectively).

**Conclusions:**

Education level and occupation are risk factors related to incidences of vaginitis in pregnant women. The bacteria-related vaginitis is a major reason of perinatal mortality.

## INTRODUCTION

During pregnancy, alterations in estrogen and progesterone levels induce physiological changes, such as PH values, in the lower genital tract of pregnant women.^1^-^3^ Such physiological changes will result in vaginal mucosa congestion and hypertrophy, which benefit growth of anaerobic bacteria and other pathogenic microorganisms within the vagina.^4^-^7^ In addition, cervical gland hypertrophy, proliferation of cervical cells, decreases in B lymphocyte numbers change the local immune environments of cervix and vagina.^8^ Increasing opportunities of infection will lead to inflammation in the vagina and cervix, therefore increasing the risk of fetus or neonate death and leading to higher perinatal mortality.

Perinatal mortality refers to the death of a fetus or neonate and is the basis to calculate the perinatal mortality rates.^9^,^10^ It has been reported that bacterial vaginosis increases the incidence of preterm birth in pregnant women and that oral clindamyc treatment reduces premature rates related to bacterial vaginosis.^11^ Svare et al. reported that for women of less than 20 week pregnancy in Denmark, vaginosis was an independent risk factor for premature, low birth weight neonates, and chorioamnionitis.^12^ Mijovic G et al. reported that earlier diagnosis of vaginal infections and timely treatments significantly reduced morbidity and mortality of perinatal newborn.^13^

Currently in China, only about 11.4% of infection cases are diagnosed. The detection rates of *trichomonas vaginitis* infection are about 1.2-2.1%.^14^ The detection rates of bacterial vaginosis in pregnant women are about 10-50%.^15^ Education level and occupation are two important factors related with vaginosis.^15^,^16^ However, whether education level and occupation are risk factor for vaginosis in pregnancy is not clear. Moreover, correlation between the vaginal infection rates with pathogenic microorganisms and the adverse perinatal outcome in China is still not clear. Especially in economically poor areas of China, reproductive tract infections during pregnancy lead to miscarriage, premature birth, premature rupture of membranes, amniotic fluid infections, neonatal pneumonia, neonatal sepsis, low birth-weight neonates, neonatal jaundice, chorioamnionitis, and postpartum endometritis. However, the correlation between the reproductive tract infections during pregnancy and the perinatal mortality is rarely reported.

In this study, the correlation between education levels and occupations of pregnant women and incidence of vaginitis for pregnant women was determined. Correlation of vaginitis during pregnancy and perinatal mortality rate was also investigated.

## METHODS

### Patients

A total of 319 women (Table-I) of early pregnancy or mid-pregnancy in Lishui region, Zhejiang province, China were enrolled from Jan. 2011 to Sep. 2013. Prior written and informed consent were obtained and the study was approved by the ethics review board of Lishui Maternity and Children Hospital, China. Six specimens were collected from posterior fornix of every pregnant woman (both asymptomatic and symptomatic) and then cultured for identification of *Neisseria gonorrhoeae*, intestinal bacteria, general bacteria, fungi, mycoplasma, and chlamydia, respectively. In all 1914 specimens collected from posterior fornix were used in this study. The study was approved by the ethics review board of Lishui Maternity and Children Hospital.

**Table-I:** Information of pregnant women.

Items (n = 319)	Ranges	Mean
Ages	20-37	26
Weights	38-108	51
Examination times before delivery	3-6	5.2
Body Mass Index	15.63-36.63	22.16

### Microorganism examination

**Neisseria gonorrhoeae** was cultured by inoculating specimens onto agar plates. Intestinal bacteria were cultured and identified using the semi-automatic bacterial identification instrument (bioMerieuxsa, Marcyletoile, French). Other kinds of bacteria were cultured by inoculating specimens into blood agar plates. Fungi were cultured by inoculating specimens into the sand paul medium. Mycoplasma was cultured and indentified using the isolation kit purchased from Zhuhai Dier bio-engineering Co., Ltd (Zhuhai, China). Chlamydia antigen was measured by *Chlamydia trachomatis* gold standard detection kit purchased from Lanzhou biological Technology Co., Ltd (Lanzhou, China). Genital tract secretion smear were made and identified.

***Follow-up investigation of*** adverse pregnancy outcomes: Patients were followed-up for 12 months. Perinatal death caused by infection (e.g. perinatal death due to chorioamnionitis or vaginitis induced preterm labor) was recorded. Perinatal mortality was calculated based on the number of perinatal death.

### Statistical analyses

SPSS 16.0 software (SPSS, Inc., Chicago, IL, USA) was used for statistical analysis. Data was presented as the mean ± standard error of the mean (SEM). Single factor analysis was performed for each factor and analyzed by the logistic regression model. P < 0.05 was considered as statistically significant differences.

## RESULTS

**Fig.1:**
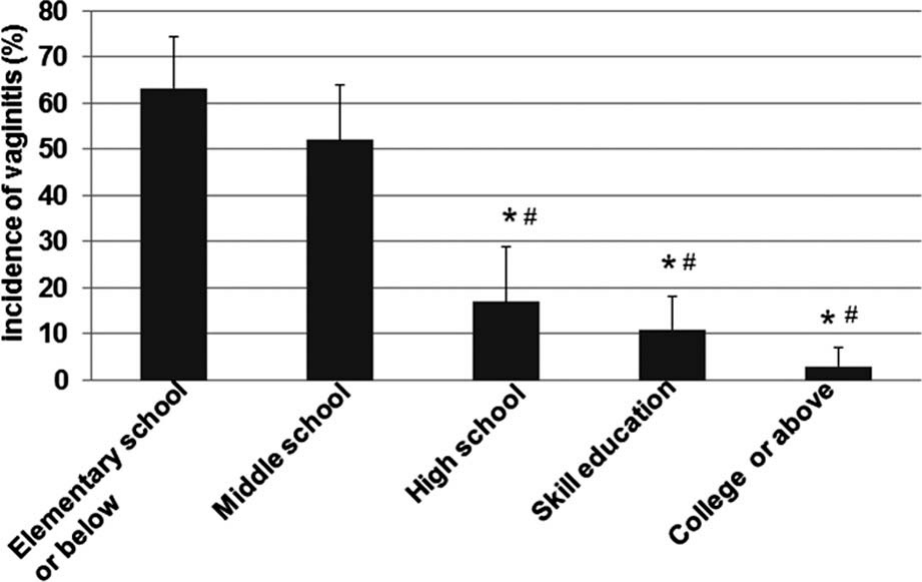
Incidences of vaginitis in pregnant women with different education levels. Vaginitis incidences of pregnant women with different education in Lishui region, Zhejiang province, China were investigated. Six specimens were collected from posterior fornix of every pregnant woman and then cultured for identification of Neisseria gonorrhoeae, intestinal bacteria, general bacteria, fungi, mycoplasma, and chlamydia, respectively. The data was expressed as mean ± SEM. P <0.05 was considered as statistically significant differences. *, P<0.05 compared with the “Elementary school or below” group. #, P < 0.05 compared with the “middle school” group.

To determine if education level is a risk factor related to incidence of vaginitis for pregnant women, vaginal secretions were collected from these pregnant women enrolled in this study and were cultured for identification of possible pathogenic microorganisms. The relationship between vaginitis incidences and education level of these pregnant women were analyzed. As shown in Fig.1, the pregnant women in the “elementary school or below” group and the “middle school” group had significant higher incidence of vaginitis (63.1 ± 10.6 and 52 ± 12.3, respectively) in comparison with the pregnant women in the groups of “high school”, “skill education”, and “college or above” (17.4 ± 11.9, 11.7 ± 8.3, and 3.7 ± 4.1, respectively). These results suggest that education level is a risk factor related to incidence of vaginitis in pregnant women.

**Fig. 2:**
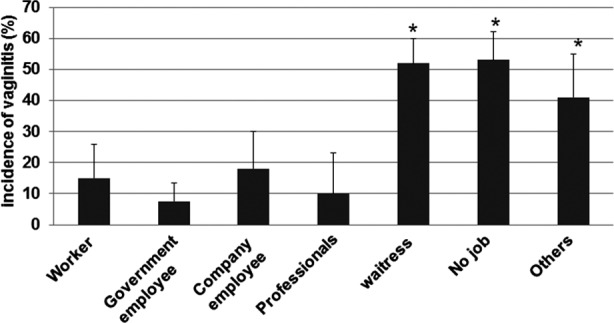
Incidences of vaginitis in pregnant women with various occupations. Vaginitis incidences of pregnant women with different occupations in Lishui region, Zhejiang province, China were investigated. Six specimens were collected from posterior fornix of every pregnant woman and then cultured for identification of Neisseria gonorrhoeae, intestinal bacteria, general bacteria, fungi, mycoplasma, and chlamydia, respectively. The data was expressed as mean ± SEM. P < 0.05 was considered as statistically significant differences. *, P < 0.05 compared with the “company employee” group.

In this study, the relationship between vaginitis incidences and occupation of these pregnant women were also analyzed. As shown in Fig.2, the vaginitis incidences of the pregnant women in the groups of “Worker”, “Government employee”, “Company employee”, and “Professionals” were obviously lower (15.2 ± 10.2, 7.5 ± 6.3, 18.1 ± 12.3, and 9.7 ± 3.1, respectively) than the vaginitis incidences in the other three groups (52.5 ± 8.2, 53.1 ± 9.3, and 41.4 ± 14.1, respectively). These results suggest that occupation is also a risk factor related to incidence of vaginitis.

**Fig.3:**
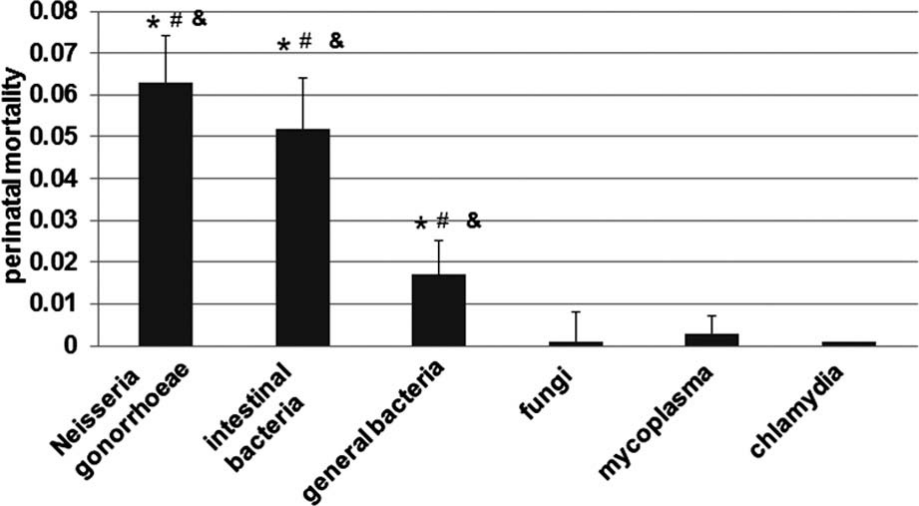
The adverse pregnancy outcomes related to pregnant women with vaginitis resulted from different infections. The perinatal mortalities were investigated. The data was expressed as mean ± SEM. P <0.05 was considered as statistically significant differences. *,P < 0.05 compared with the “fungi” group. ^#^, P < 0.05 compared with the “mycoplasma” group. ^&^, P < 0.05 compared with the “chlamydia” group.

To determine if vaginitis is related to adverse pregnancy outcomes, perinatal death caused by infection (e.g. perinatal death due to chorioamnionitis or vaginitis induced preterm labor) was recorded. And then perinatal mortality was calculated based on the number of perinatal death. As shown in Fig.3, the women with infections of bacteria, including *Neisseria gonorrhoeae*, intestinal bacteria, and general bacteria were related to higher perinatal mortalities (0.063 ± 0.011, 0.052 ± 0.012, and 0.017 ± 0.008, respectively) than women with infections of fungi, mycoplasma, and Chlamydia (0.002 ± 0.007, 0.003 ± 0.004, and 0.001 ± 0.001, respectively). These results suggest that vaginitis resulted from bacteria is a major reason of perinatal mortalities.

## DISCUSSION

Microorganism infection of reproductive tract during pregnancy is a major cause of vaginitis. Microorganism infection is related to women’s behaviors. This study showed that education level and occupation are two risk factors related to incidence of vaginitis in pregnant women. Our result was consistent with a previous study by Zhang et al.^16^ Public health is often related to people’s education and their occupations.^17^,^18^ For example, occurrences of some endemic and emerging diseases, such as SARS, H5N1, and H1N1 influenza, are related to behaviors of humans and their education and occupations.^16^ Education level and condom use are protective factors of human papilloma virus infection in Urumqi, China, but occupation is a risk factor for human papilloma virus infection of women in this region.^18^

The bacterial infections in the lower female reproductive tract are a common reason of reproductive tract infection resulting in adverse perinatal outcome. It is reported that vaginitis in pregnancy is related to adverse perinatal outcome.^19^,^20^ Consistently, in this study, the women with infections of bacteria, such as *Neisseria gonorrhoeae*, intestinal bacteria, and general bacteria, had higher perinatal mortalities (0.063 ± 0.011, 0.052 ± 0.012, and 0.017 ± 0.008, respectively) than women with infections of fungi, mycoplasma, and Chlamydia (0.002 ± 0.007, 0.003 ± 0.004, and 0.001 ± 0.001, respectively). These results suggest that vaginitis resulted from bacteria is a major reason of perinatal mortalities when compared with vaginitis caused by fungi or other types of microorganisms. The fetus is protected from microorganism infection by the cervix, which controls and limits microbial infection by production of immune cytokines, and antimicrobial molecules.^21^ If this barrier is affected, bacteria may enter the uterine cavity, leading to adverse perinatal outcome. Therefore, improving women’s living ways and knowledge regarding reproductive health issues will help decrease the incidences of vaginitis and reduce adverse pregnancy outcomes.

This study mainly has two limitations. First, this report only studied the factors of education level and occupation in occurrence of vaginitis. The other risk factors for vaginitis in pregnancy, such as race/ethnicity, age, low income, sexual practices, smoking, etc., were not analyzed. Second, this study only reported the relationship between occurrence of vaginitis in pregnancy and perinatal mortality rates. The relationship between occurrence of vaginitis in pregnancy and other adverse perinatal outcomes (such as neonatal pneumonia, neonatal sepsis, low birth weight babies, neonatal jaundice, chorioamnionitis, postpartum endometritis, etc.) were not investigated.

In conclusion, our results showed that education level and occupation were risk factors related to incidences of vaginitis in pregnant women. The bacteria-related vaginitis was a major reason of perinatal mortalities.
